# Crude Drugs for Clearing Heat Contain Compounds Exhibiting Anti-Inflammatory Effects in Interleukin-1β-Treated Rat Hepatocytes

**DOI:** 10.3390/molecules30020416

**Published:** 2025-01-19

**Authors:** Airi Fujii, Saki Onishi, Nodoka Watanabe, Mizuki Kajimura, Kentaro Ito, Keita Minamisaka, Yuto Nishidono, Saki Shirako, Yukinobu Ikeya, Mikio Nishizawa

**Affiliations:** 1 Department of Medical Biosciences, Faculty of Life Sciences, Ritsumeikan University, Kusatsu 525-8577, Shiga, Japan; fujii-ai@fc.ritsumei.ac.jp (A.F.); sshirako@fc.ritsumei.ac.jp (S.S.); 2 Research Organization of Science and Technology, Ritsumeikan University, Kusatsu 525-8577, Shiga, Japan; nisidono@fc.ritsumei.ac.jp (Y.N.); y-ikeya@daiichi-cps.ac.jp (Y.I.); 3 Faculty of Pharmacy, Daiichi University of Pharmacy, Fukuoka 815-8511, Fukuoka, Japan; 4 Department of Biology, Faculty of Mathematics and Natural Sciences, Universitas Brawijaya, Malang 65113, East Java, Indonesia

**Keywords:** clearing heat, *Seinetsu*, *Yakuno*, inflammation, nitric oxide, cytokine

## Abstract

Traditional Japanese medicines, i.e., Kampo medicines, consist of crude drugs (mostly plants) that have empirical pharmacological functions (‘*Yakuno*’ in Japanese), such as clearing heat. Crude drugs with cold properties, such as *Phellodendron* bark, have the empirical function of clearing heat as they cool the body. Because we found that anti-inflammatory compounds were present in several crude drugs for clearing heat, it is speculated that the empirical function of clearing heat may be linked to anti-inflammatory activities. When 10 typical crude drugs were selected from 22 herbal crude drugs for clearing heat, we identified anti-inflammatory compounds in five crude drugs, including *Phellodendron* bark. In this study, the other crude drugs were extracted and partitioned with ethyl acetate (EtOAc) and *n*-butanol to obtain three crude fractions. All the EtOAc-soluble fractions, except that from *Forsythia* fruits, inhibited interleukin (IL)-1β-induced nitric oxide (NO) production in primary-cultured rat hepatocytes. Anti-inflammatory compounds were identified from these EtOAc-soluble fractions: baicalein from *Scutellaria* roots, (−)-nyasol from *Anemarrhena* rhizomes, and loniflavone from *Lonicera* leaves and stems. (+)-Phillygenin was purified from *Forsythia* fruits by removing cytotoxic oleanolic and betulinic acids. These compounds suppressed the production of NO and cytokines in hepatocytes. Anti-inflammatory compounds were not purified from the EtOAc-soluble fraction of *Rehmannia* roots because of their low abundance. Collectively, these findings indicate that anti-inflammatory compounds are present in all 10 crude drugs for clearing heat, confirming that these anti-inflammatory compounds in crude drugs provide the empirical functions for clearing heat. Other empirical functions of Kampo medicine can also be explained by modern pharmacological activities.

## 1. Introduction

Medicinal plants that grow in China, Korea, and Japan, have been used for traditional Chinese medicine (TCM) and traditional Japanese medicine, i.e., Kampo medicine, which was independently developed from traditional Chinese medicine during the 18th century [1,2]. Most ‘crude drugs’ used for Kampo medicine are specific parts of medicinal plants [3]. A Kampo formula consists of crude drugs, each of which is classified using four properties (cold, cool, warm, and heat) and five tastes (sour, bitter, sweet, spice, and salt) based on the Yin–yang and Five elements theories [1,4]. Crude drugs have empirical pharmacological functions (known as ‘*Yakuno*’ in Japanese), such as clearing heat to cool the body’s heat (‘*Seinetsu*’), replenishing blood to improve blood deficiency (‘*Hoketsu*’), and pus discharge (‘*Haino*’) [1,5]. Crude drugs are classified into 13 categories on the basis of their empirical functions in the KEGG BRITE database [6]. Although many constituents of crude drugs have been reported to date, there are few reports discussing the mechanisms of empirical functions, as well as the relationships between the constituents and empirical functions of the crude drugs used for Kampo medicine, from a modern pharmacological perspective.

Crude drugs with cold properties have the empirical function of clearing heat as they cool the body [1]. According to the KEGG BRITE database, 25 crude drugs for clearing heat are known, and 22 are derived from medicinal plants [6]. When 10 typical crude drugs were selected (Table 1), we previously purified and identified anti-inflammatory compounds from five crude drugs, i.e., *Phellodendron amurense* bark, *Coptis chinensis* rhizomes, *Paeonia suffruticosa* root bark, and *Lonicera japonica* flowers and buds [7,8,9]. In addition, the anti-inflammatory effects of the constituents of *Gardenia jasminoides* fruits, i.e., genipin and geniposide, were previously reported [10,11,12]. These compounds exhibited anti-inflammatory effects in primary-cultured rat hepatocytes [7,8,9,10] or the macrophage line RAW264.7 [11,12]. Accordingly, it is expected that the empirical function of clearing heat is linked to anti-inflammatory compounds in crude drugs.

The proinflammatory mediator nitric oxide (NO) is produced in hepatocytes and macrophages in response to interleukin 1β (IL-1β) and lipopolysaccharide (LPS), respectively [13]. An anti-inflammatory compound that was added to the medium suppressed NO production and downregulated proinflammatory genes in both hepatocytes and macrophages [14,15]. Therefore, the inhibition of NO production is an inflammatory response. To date, we have used primary-cultured rat hepatocytes treated with IL-1β to monitor the anti-inflammatory effects of crude drugs and their constituents and to compare their activities [7,8,9,10].

To verify the hypothesis that the crude drugs for clearing heat contain anti-inflammatory compounds, we investigated their ability to clear heat, which we did not previously examine (Table 1). We attempted to purify anti-inflammatory compounds from the five crude drugs by monitoring IL-1β-induced NO production in primary-cultured rat hepatocytes. Lastly, we discuss approaches to elucidate the empirical pharmacological function of crude drugs using modern pharmacological methods.

## 2. Results

### 2.1. Extraction and Fractionation of Crude Drugs

**Scheme 1:**
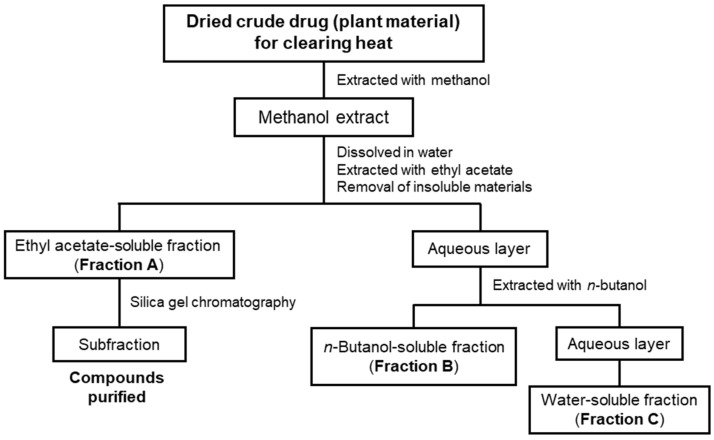
A flowchart showing the purification of compounds from crude drugs for clearing heat. Methanol extracts from crude drugs were fractionated into three fractions to purify the compounds by silica gel chromatography, preparative thin-layer chromatography (TLC), and so forth. Each fraction or compound was subjected to measurements of the nitric oxide (NO) production in interleukin (IL)-1β-treated hepatocytes.

As mentioned above, we previously isolated and identified anti-inflammatory compounds from four crude drugs for clearing heat, as shown in Table 1, i.e., *Phellodendron* bark, *Coptis* rhizome, Moutan bark, and *Lonicera* flower are shown [7,8,9]. Therefore, we extracted other crude drugs for clearing heat with methanol and successively fractionated the extract with ethyl acetate (EtOAc) and *n*-butanol into three fractions (Scheme 1) in accordance with a previously published method [9].

The yields of the extraction and partitioning ratios of the fractions are shown in Table 2. Our previous data are also presented [7,8,9]. The extraction yields ranged from 8.93 to 34.6%. Because Fractions A, B, and C include hydrophobic, amphipathic, and hydrophilic compounds, respectively [9], the partitioning ratios of the fractions differ depending on the crude drug and the parts of the plants.

### 2.2. Effects of the Fractions on the Suppression of NO Production in Hepatocytes

Next, the effect of each fraction on NO production was evaluated via IL-1β-treated rat hepatocytes. We determined their half-maximal inhibitory concentration (IC_50_) values, according to a previously established method [14]. As shown in Table 2, Fraction A of the crude drugs, excluding *Forsythia* fruits, presented lower IC_50_ values than Fractions B and C did. The results suggest that Fraction A of the crude drugs (*Gardenia* fruit, *Scutellaria* root, *Anemarrhena* rhizome, *Lonicera* leaf and stem, and *Rehmannia* root) contains hydrophobic compounds that suppress NO production in hepatocytes. Therefore, we attempted to purify anti-inflammatory compounds from these crude drugs (see below).

In contrast, an IC_50_ value could not be calculated when Fraction A of *Forsythia* fruits was added to the medium, because many hepatocytes detached from the dish bottom due to its cytotoxicity. The cytotoxic compounds in this fraction may mask the activity of compounds that suppress NO production. Therefore, we also tried to purify compounds from *Forsythia* fruit Fraction A.

### 2.3. Purification of Anti-Inflammatory Compounds from Crude Drugs

#### 2.3.1. Genipin and Geniposide of *Gardenia* Fruits

Although there are reports that genipin and geniposide from *Gardenia jasminoides* fruits have anti-inflammatory effects [10,11,12], information about the fractionation of active compounds in *Gardenia* fruit extracts is not available. Following extraction and fractionation into three crude fractions (Table 2), only Fraction A suppressed NO production in the IL-1β-treated hepatocytes. The IC_50_ value of genipin (aglycone) for the suppression of NO production was 4.40 ± 0.85 μM, whereas geniposide (glycoside) did not inhibit NO production up to a final concentration of 800 μM. HPLC analysis using genipin as a standard demonstrated that genipin was present in Fraction A, indicating that genipin is at least responsible for the activity of *Gardenia* fruits.

#### 2.3.2. Purification of Baicalin from *Scutellaria* Roots

First, we purified the constituents in Fraction A from a *Scutellaria* root extract, as described in the Materials and Methods. Guided by NO assays, we purified a biologically active compound from the subfraction that showed high activity against NO production in hepatocytes (Supplementary Materials Data S1.1.). This compound (5.14 mg), which was designated as Compound **1**, was identified by nuclear magnetic resonance (NMR) spectroscopy analysis.

**Figure 1:**
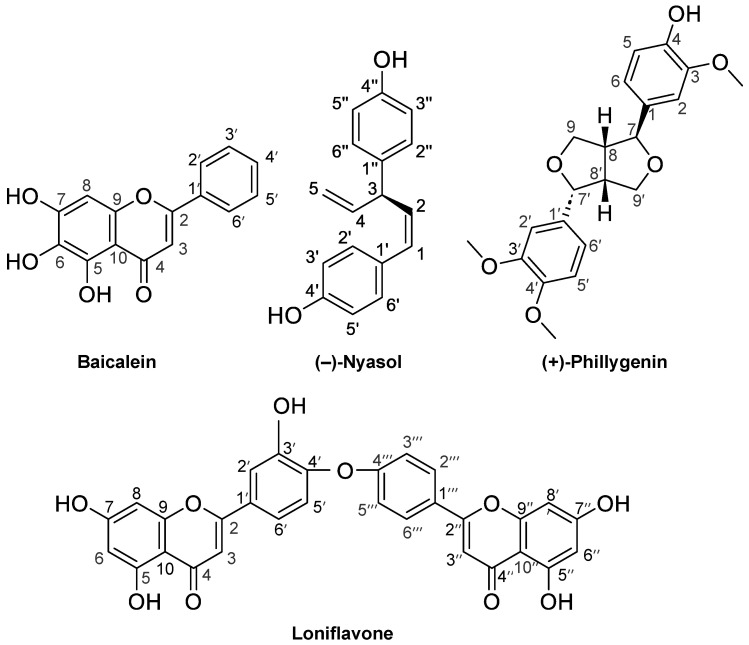
The chemical structures of the compounds purified in this study. Baicalein (Compound **1**) from *Scutellaria* roots, (−)-nyasol (**2**) from *Anemarrhena* rhizomes, loniflavone (**3**) from *Lonicera* leaves and stems, and (+)-phillygenin (**4**) from *Forsythia* fruits.

Compound **1**: Yellow powder. ^1^H NMR [500 MHz, (CD_3_)_2_SO, ppm] δ 8.06 (2H, d, *J* = 2.0 Hz, H-2′, H-6′), 7.59 (3H, m, H-3′, H-4′, H-5′), 6.95 (1H, s, H-3), 6.64 (1H, s, H-8); ^13^C NMR [125 MHz, (CD_3_)_2_SO, ppm] δ 182.0 (C-4), 162.7 (C-7), 153.6 (C-2), 149.7 (C-9), 146.8 (C-5), 131.8 (C-4′), 130.8 (C-1′), 129.2 (C-6), 129.0 (C-3′, C-5′), 126.2 (C-2′, C-6′), 104.4 (C-3), 104.1 (C-10), 93.9 (C-8). This compound was identified as baicalein on the basis of ^1^H and ^13^C NMR spectral analysis by comparison with previously published results [16], (see Figure 1).

**Figure 2:**
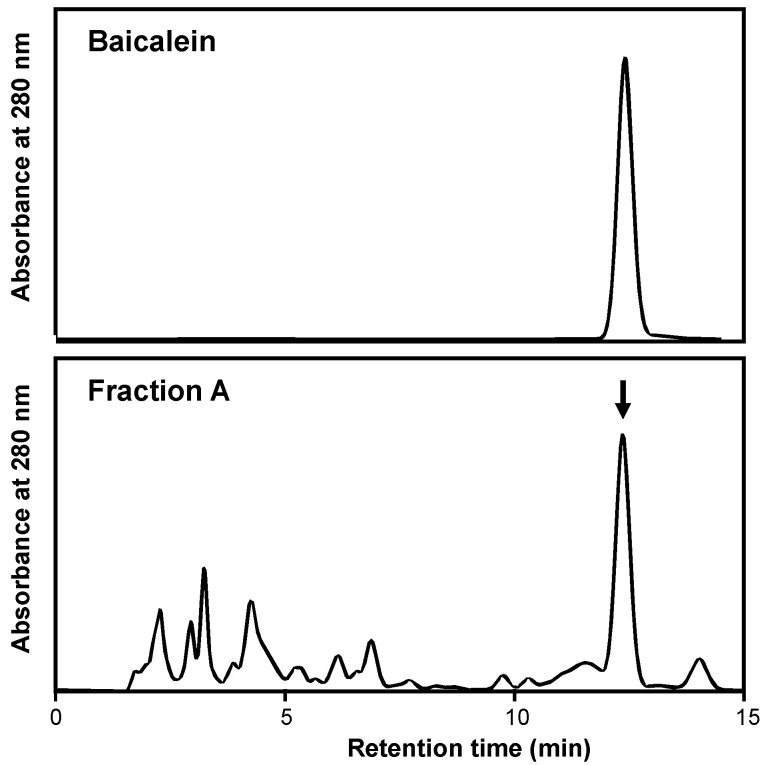
HPLC chromatograms of baicalein (standard; upper) and Fraction A from the *Scutellaria* root extract (lower). HPLC was used for this analysis, as described in Section 4. The arrow indicates the peak of baicalein.

The baicalein in a *Scutellaria* root extract was analyzed. The HPLC chromatogram of Fraction A showed an independent peak (retention time, 12.4 min), which corresponded to that of baicalein (Figure 2). The content of baicalein was subsequently estimated by HPLC and calculated as 22.5% in Fraction A and 3.58% in the extract (Supplementary Materials Data, S2.1.). Given that the extraction yield was 34.6% (Table 1), the content of baicalein in *Scutellaria* roots was calculated as 1.24, i.e., 12.4 mg/g of the plant material, which was comparable to the reported values ranging from 5.747 to 15.449 mg/g [17].

**Figure 3:**
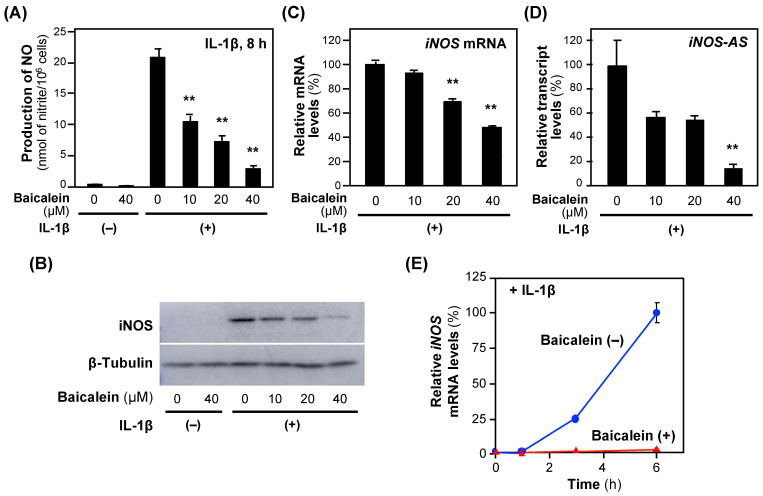
The effects of baicalein on the expression of the *iNOS* gene in hepatocytes. (**A**) The effects of baicalein on NO production. Baicalein and IL-1β were added to the medium of primary-cultured rat hepatocytes and incubated for 8 h. The nitrate concentrations in the medium were measured as NO. Cytotoxicity was not observed at the concentrations applied. (**B**) An immunoblot analysis of the iNOS protein. Hepatocyte extracts were prepared from hepatocytes in (**A**) and analyzed by immunoblotting to detect the iNOS (130 kDa) and the internal control β-tubulin (55 kDa). (**C**) The levels of *iNOS* mRNA and (**D**) *iNOS* antisense transcript (*iNOS-AS*). Total RNA was extracted 3 h after the addition of baicalein and subjected to quantitative reverse transcription–polymerase chain reaction (RT–qPCR). The level of each mRNA was measured and normalized to the elongation factor 1α (*Ef1a*) mRNA level. Relative mRNA levels (%) are presented as the means ± SDs (*n* = 3) when the measured mRNA level was set as 100% in the presence of IL-1β alone. ** *p* < 0.01 versus IL-1β alone. (**E**) The time course of the *iNOS* mRNA levels. After the addition of 40 μM baicalein (*t* = 0 h), total RNA was extracted at the indicated times and subjected to RT–qPCR. Relative mRNA levels (%) were normalized to *Ef1a* mRNA levels and are presented as the means ± SDs (*n* = 3), and the mRNA level measured 6 h after the addition of IL-1β was set as 100%. ** *p* < 0.01 versus IL-1β alone.

Next, the effects of baicalein on NO production in hepatocytes were examined. When baicalein was added to the medium of the rat hepatocytes together with IL-1β, the NO concentration decreased in a concentration-dependent fashion without showing cytotoxicity, i.e., <5% of the activity of the whole-cell extract (Figure 3). The calculated IC_50_ value of baicalein for suppressing NO production was 16.8 ± 7.93 μM.

Next, we investigated the *iNOS* gene expression. Baicalein reduced both iNOS protein and *iNOS* mRNA levels (Figure 3B,C), suggesting that the *iNOS* gene expression is regulated at a transcriptional step. Because a natural antisense transcript (*iNOS-AS*) transcribed from the *iNOS* gene interacts with and stabilizes *iNOS* mRNA [18], we analyzed the effects of baicalein on *iNOS-AS* expression. Baicalein inhibited *iNOS-AS* in a concentration-dependent manner (Figure 3D). The *iNOS* mRNA level increased in the presence of IL-1β, whereas it was inhibited by baicalein for 6 h after the addition of IL-1β (Figure 3E). Similarly to chlorogenic acid [9], baicalein inhibits the induction of the *iNOS* gene expression at both the transcriptional and post-transcriptional levels.

#### 2.3.3. Purification of (−)-Nyasol from *Anemarrhena* Rhizomes

To purify and identify the constituents of the *Anemarrhena* rhizome extract, Fraction A was fractionated into subfractions via silica gel column chromatography and preparative thin-layer chromatography (TLC). We purified an active compound from the subfraction that showed high activity against NO production. Lastly, Compound **2** (18.5 mg) was isolated (Supplementary Materials Data, S1.2.) and identified by NMR spectroscopy and mass spectrometry (MS) analysis.

Compound **2**: Pale yellow oil. [*α*${]}_{D}^{23}$ −72.8° (*c* 0.257, CHCl_3_); EI-MS *m*/*z* (%): 252 (M^+^, 100), 237 (20), 158 (32), 145 (29), 107 (28) HR-EI-MS *m*/*z* 252.1156 (M^+^) (calculated for C_17_H_16_O_2_: 252.1150); ^1^H NMR (400 MHz, CD_3_OD, ppm) *δ* 7.16 (2H, d, *J* = 8.8 Hz, H-2′,6′), 7.09 (2H, d, *J* = 8.0 Hz, H-2″,6″), 6.78 (2H, d, *J* = 8.8 Hz, H-3′,5′), 6.77 (2H, d, *J* = 8.0 Hz, H-3″,5″), 6.51 (1H, d, *J* = 11.6 Hz, H-1), 6.00 (1H, ddd, *J* = 17.2, 10.8, 6.4 Hz, H-4), 5.66 (1H, dd, *J* = 11.6, 10.0 Hz, H-2), 5.16(2H, ddd, *J* = 1.2, 4.8, 11.7 Hz, H-5), 4.48 (1H, dd, *J* = 10.0, 6.4 Hz, H-3). ^13^C NMR (100 MHz, CD_3_OD, ppm) *δ* 154.61 (C-4′), 154.14 (C-4″), 140.77 (C-4), 135.66 (C-1″), 131.78 (C-2), 130.11 (C-2′ and 6′), 129.91 (C-1′), 128.97 (C-2″ and 6″), 128.68 (C-1), 115.47 (C-3″ and 5″), 115.20 (C-3′,5′ and 5), 46.69 (C-3). These data were almost the same as the previously published results for (−)-nyasol (=*cis*-hinokiresinol) isolated from *Anemarrhena asphodeloides* rhizomes, on the basis of ^1^H and ^13^C NMR spectral analysis and optical rotation [α${]}_{D}$ −67° (*c* 0.1, C_3_H_6_O) [19,20]. Therefore, this compound was identified as (−)-nyasol.

To determine the (−)-nyasol content in *Anemarrhena* rhizome extract, HPLC analysis was performed. The HPLC results revealed several independent peaks in the chromatogram of Fraction A, and the (−)-nyasol content was subsequently estimated; it was 5.56% in Fraction A and 0.773% in the extract (Supplementary Materials Data, S2.2.).

**Figure 4:**
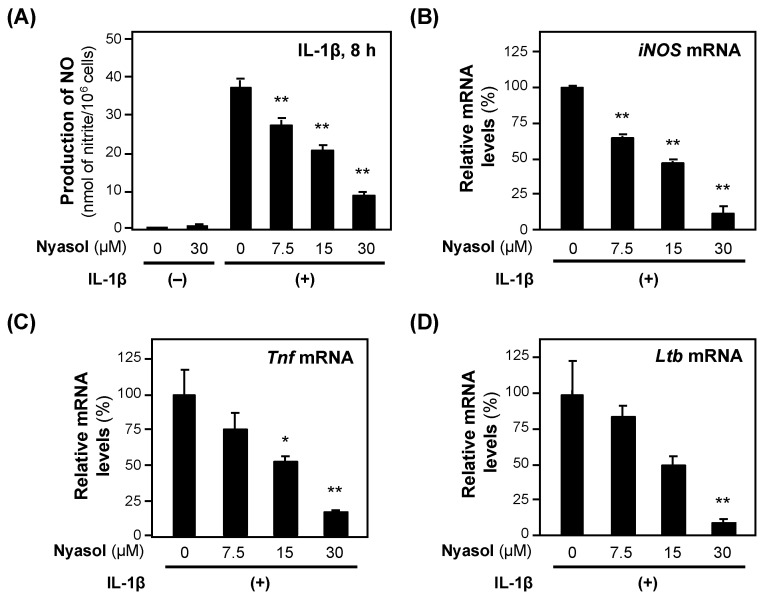
The effects of (−)-nyasol on the expression of proinflammatory genes. (**A**) NO levels. (−)-Nyasol and IL-1β were added to the hepatocyte medium and incubated. Cytotoxicity was not observed at the concentrations applied. After 4 h, the total RNA was extracted and subjected to RT–qPCR. Each mRNA level was measured in triplicate and normalized to the *Ef1a* mRNA level: (**B**) *iNOS* mRNA; (**C**) *Tnf* mRNA; and (**D**) lymphotoxin β (*Ltb*) mRNA. The relative mRNA levels (%) are presented as the means ± SDs (*n* = 3) when the mRNA level was set at 100% in the presence of IL-1β alone. * *p* < 0.05 and ** *p* < 0.01 versus IL-1β alone.

When (−)-nyasol was added to the medium of the rat hepatocytes together with IL-1β, the NO concentration decreased in a concentration-dependent manner without showing cytotoxicity, i.e., <5% of the activity of the whole-cell extract (Figure 4). The calculated IC_50_ value of (−)-nyasol for the suppression of NO production was 18.3 ± 2.99 μM. (−)-Nyasol significantly decreased the levels of *iNOS* mRNA. Furthermore, (−)-nyasol significantly reduced the levels of mRNAs encoded by proinflammatory genes, such as tumor necrosis factor α (*Tnf*) and lymphotoxin β (*Ltb*) mRNAs (Figure 4).

#### 2.3.4. Purification of Loniflavone from *Lonicera* Leaves and Stems

To purify the anti-inflammatory compounds, Fraction A of the extract from *Lonicera* leaves and stems was purified, as described in Materials and Methods. Subfractions that suppressed NO production in hepatocytes were further purified to obtain Compound **3** (5.73 mg) (Supplementary Materials Data, S1.3.).

Compound **3**: Pale yellow powder. ^1^H NMR [400 MHz, (CD_3_)_2_CO, ppm] *δ* 12.92 (1H, s, H-5), 12.91 (1H, s, H-5″), 8.03 (2H, d, *J* = 9.1 Hz, H-2‴/H-6‴), 7.89 (1H, dd, *J* = 8.5, 2.1 Hz, H-6′), 7.87 (1H, d, *J* = 2.1 Hz, H-2′), 7.24 (1H, d, *J* = 8.5 Hz, H-5′), 7.10 (2H, d, *J* = 8.8 Hz, H-3‴/H-5‴), 6.70 (1H, s, H-3″), 6.69 (1H, s, H-3), 6.52 (1H, d, *J* = 2.1 Hz, H-8), 6.51 (1H, d, *J* = 2.1 Hz, H-8″), 6.23 (1H, d, *J* = 1.8 Hz, H-6), 6.22 (1H, d, *J* = 2.1 Hz, H-6″). ^13^C NMR [100 MHz, (CD_3_)_2_CO, ppm] *δ* 182.2 (C-4), 182.2 (C-4′), 164.2 (C-7), 164.2 (C-7′), 163.5 (C-2), 163.1 (C-2′), 162.5 (C-5), 162.5 (C-5′), 161.1 (C-4‴), 158.0 (C-9″), 157.9 (C-9), 153.1 (C-3′), 142.4 (C-4′), 128.4 (C-2‴/C-6‴), 125.4 (C-1‴), 125.1 (C-6′), 123.7 (C-1′), 120.9 (C-2′), 118.2 (C-5′), 116.6 (C-3‴/C-5‴), 104.6 (C-10″), 104.5 (C-10), 104.3 (C-3″), 104.0 (C-3), 99.0 (C-6), 99.0 (C-6″), 94.0 (C-8), 94.0 (C-8″). This compound was identified as loniflavone (Figure 1) on the basis of ^1^H and ^13^C NMR spectral analysis in comparison with a previous report on loniflavone isolated from *Caesalpinia pyramidalis* leaves [21].

We first measured the NMR spectrum of Compound **3** in (CD_3_)_2_SO. ^1^H NMR [400 MHz, (CD_3_)_2_SO, ppm] *δ* 12.88 (1H, s, H-5), 12.85 (1H, s, H-5″), 8.01 (2H, d, *J* = 9.8 Hz, H-2‴/H-6‴), 7.88 (1H, dd, *J* = 9.1, 2.4 Hz, H-6′), 7.88 (1H, d, *J* = 2.1 Hz, H-2′), 7.12 (1H, d, *J* = 9.1 Hz, H-5′), 6.99 (2H, d, *J* = 9.1 Hz, H-3‴/H-5‴), 6.85 (1H, s, H-3″), 6.85 (1H, s, H-3), 6.46 (1H, d, *J* = 2.1 Hz, H-8), 6.45 (1H, d, *J* = 2.1 Hz, H-8″), 6.16 (1H, d, *J* = 2.1 Hz, H-6), 6.15 (1H, d, *J* = 2.1 Hz, H-6″). ^13^C NMR [100 MHz, (CD_3_)_2_SO, ppm] *δ* 182.3 (C-4), 182.3 (C-4′), 164.8 (C-7), 164.7 (C-7′), 163.6 (C-2), 163.2 (C-2′), 162.0 (C-5), 161.9 (C-5′), 161.3 (C-4‴), 157.9 (C-9″), 157.8 (C-9), 153.9 (C-3′), 142.1 (C-4′), 129.0 (C-2‴/ C-6‴), 125.9 (C-6′), 124.9 (C-1‴), 122.8 (C-1′), 121.8 (C-2′), 118.4 (C-5′), 116.6 (C-3‴/C-5‴), 104.5 (C-10″), 104.3 (C-10), 104.3 (C-3″), 104.1 (C-3), 99.4 (C-6), 99.4 (C-6″), 94.6 (C-8), 94.6 (C-8″).

**Figure 5:**
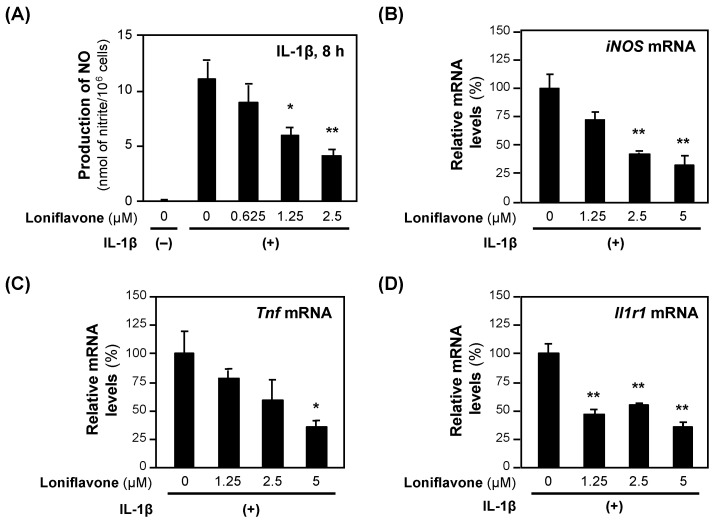
The effects of loniflavone on the expression of proinflammatory genes. (**A**) Decreased NO production by baicalein in hepatocytes. Loniflavone and IL-1β were added to the medium of primary-cultured rat hepatocytes and incubated for 8 h until the NO concentration was measured. Cytotoxicity was not observed at the concentrations applied. (**B**–**D**) mRNA levels in hepatocytes. After incubation with loniflavone and IL-1β, the total RNA was extracted and subjected to RT–qPCR. The levels of each mRNA were measured in triplicate and normalized to the *Ef1a* mRNA level. The relative mRNA levels (%) of *iNOS* (**B**), *Tnf* mRNA (**C**), and *Il1r1* (**D**) are presented as the means ± SDs (*n* = 3), when the measured mRNA level was set at 100% in the presence of IL-1β alone. * *p* < 0.05 and ** *p* < 0.01 versus IL-1β alone.

Next, we examined the effects of loniflavone on the expression of the mRNAs encoding proinflammatory genes. Loniflavone decreased the levels of *iNOS* and *Tnf* mRNAs in a concentration-dependent manner (Figure 5). IL-1β induces the expression of type 1 interleukin 1 receptor (IL1R1), which regulates the expression of proinflammatory genes, including the *iNOS* gene [22]. When we measured the *Il1r1* mRNA levels, loniflavone significantly decreased the *Il1r1* mRNA levels (Figure 5).

### 2.4. Unsuccessful Purification of Anti-Inflammatory Compounds

Because *Rehmannia* root Fraction A strongly suppressed NO production in hepatocytes (Table 2), we attempted to purify the anti-inflammatory compounds. When Fraction A from the *Rehmannia* root extract was separated by silica gel column chromatography, the compounds were eluted stepwise using an *n*-hexane–EtOAc mixture (100:0 ---> 0:100) to provide subfractions A1 to A14. When each subfraction was added to the medium at a final concentration of 50 μg/mL, six subfractions (A2 to A7) reduced NO production to less than 50% of that in the presence of IL-1β. Furthermore, many spots were visualized by spraying *p*-anisaldehyde–sulfuric acid solution, indicating that several anti-inflammatory compounds were present in Fraction A.

Many constituents in the *Rehmannia* roots have been reported [23], and most of them are iridoid glycosides (e.g., glutinoside and catalpol) and sugars (e.g., stachyose), which seem to be partitioned to Fractions B and C, respectively. Conversely, the ratio of Fraction A was 1.71%, which was the lowest among the 10 crude drugs (Table 2). Because hydrophobic compounds are expected to be present at low abundances in Fraction A, we stopped purifying anti-inflammatory compounds from Fraction A of *Rehmannia* roots.

### 2.5. Identification of an Anti-Inflammatory Compound by Removing Masking Compounds

As shown in Table 2, Fraction A of the *Forsythia* fruit extract was cytotoxic, suggesting that the cytotoxic compounds in this fraction may mask the activity of anti-inflammatory compounds that suppress IL-1β-induced NO production. Fraction A from *Forsythia* fruits was subjected to silica gel column chromatography, and the compounds were eluted stepwise using an *n*-hexane–EtOAc mixture to provide subfractions A1 to A13. When each subfraction was added to the medium, only subfractions A3 and A4 exhibited high LDH activity.

Triterpenoids, e.g., oleanolic acid, are present in *Forsythia suspensa* fruits [24] and *Zizyphus jujuba* fruits [25], and the cytotoxicity of betulinic acid has been reported [25,26]. The subfractions were developed by TLC using the standards of oleanolic and betulinic acids. Spots with the same *R*_f_ as those of oleanolic (*R*_f_ = 0.13) and betulinic acid (*R*_f_ = 0.20) were visualized by using the *p*-anisaldehyde–sulfuric acid coloring reaction. The spots observed via TLC indicated that oleanolic and betulinic acids were present in subfractions A3 and A4.

**Figure 6:**
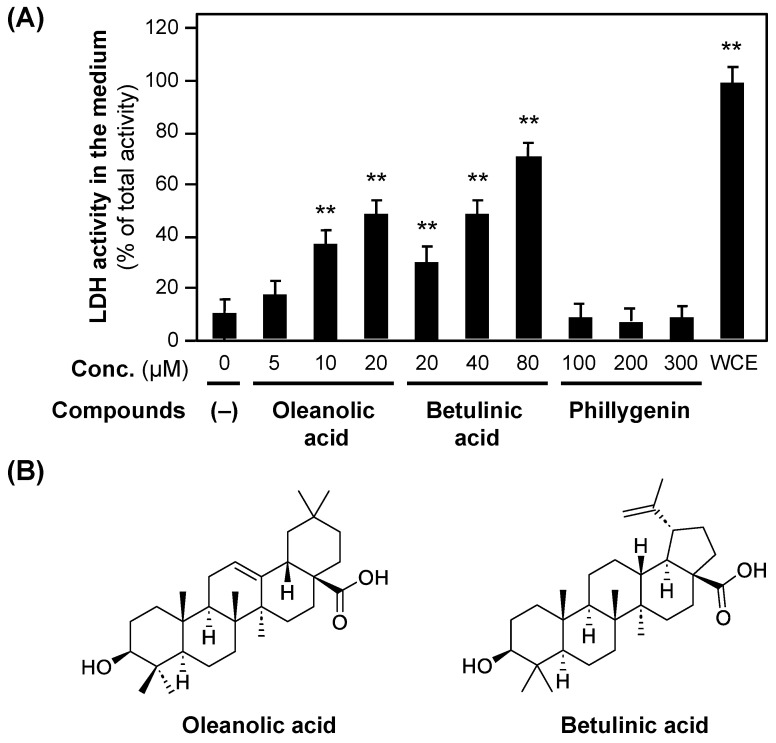
The cytotoxicity of oleanolic acid and betulinic acid. (**A**) The cytotoxicity of oleanolic acid and betulinic acid, measured by LDH activity in the hepatocyte medium. Oleanolic acid, betulinic acid, or (+)-phillygenin was added to the medium at the indicated concentrations. After incubation for 8 h, the LDH activity of the medium was measured. When the LDH activity of the whole-cell extract (WCE) of hepatocytes on a dish was set at 100%, each LDH activity was recorded. ** *p* < 0.01 versus LDH activity at 0 μM. (**B**) The chemical structures of oleanolic acid and betulinic acid.

To investigate whether these triterpenoids are cytotoxic, the LDH activity of the medium was measured in the presence of oleanolic or betulinic acid. As shown in Figure 6, both oleanolic and betulinic acids increased LDH activity in a concentration-dependent manner. In contrast, (+)-phillygenin resulted in background LDH activity at concentrations up to 300 μM, indicating no cytotoxicity.

The other subfractions, excluding A3 and A4, which did not include oleanolic or betulinic acid, were eluted by silica gel column chromatography. These subfractions were examined to determine whether they inhibited NO production in hepatocytes. One subfraction that markedly inhibited NO production was further purified to obtain a compound (174 mg) (Supplementary Materials Data S1.4.), which was designated as Compound **4** and analyzed by NMR spectroscopy and MS analyses.

Compound **4**: White powder. [*α*${]}_{D}^{20}$ +95.4° (*c* 0.763, CH_3_OH); EI–MS spectrum *m*/*z* 372.1562 (M^+^) (calculated for C_21_H_24_O_6_; 372.1573). ^1^H NMR (400 MHz, CDCl_3_, ppm) *δ* 2.92 (1H, m, H-8), 3.33 (1H, m, H-9′ ax), 3.35 (1H, m, H-8′), 3.85 (2H, m, H-9 eq, H-9′ eq), 3.87(3H, s, -OCH_3_), 3.89 (3H, s, -OCH_3_), 3.90 (3H, s, -OCH_3_), 4.13 (1H, d, *J* = 9.6 Hz, H-9), 4.42 (1H, d, *J* = 7.2 Hz, H-7), 4.87 (1H, d, J = 5.9 Hz, H-7′), 5.60 (1H, s, -OH), 6.82–6.92 (6H, m, H-2, 5, 6, 2′, 5′, 6′). ^13^C NMR (400 MHz, CDCl_3_, ppm) *δ* 50.2 (C-8′), 54.5 (C-8), 55. 9 (OCH_3_), 56.0 × 2 (-OCH_3_ × 2), 69.7 (C-9′), 71.0 (C-9), 82.1 (C-7′), 87.8 (C-7), 108.6 (C-2′), 109.0 (C-2), 111.0 (C-5′), 114.3 (C-5), 117.8 (C-6′), 119.3 (C-6), 131.0 (C-1′), 133.1 (C-1), 145.4 (C-4), 146.8 (C-4′), 148.0 (C-3), 148.9 (C-3′). The ^1^H and ^13^C NMR spectra of this compound were identical to previously published spectral data for (+)-phillygenin [27]. The EI–MS spectrum data indicated that this compound was calculated for C_21_H_24_O_6_ (372.1573), which is identical to the molecular formula of phillygenin. The optical rotation measured was close to the reported value [*α*${]}_{D}^{22}$ + 90.0° (*c* 0.2, CH_3_OH) of (+)-phillygenin [28]. Overall, we concluded that Compound **4** is (+)-phillygenin.

**Figure 7:**
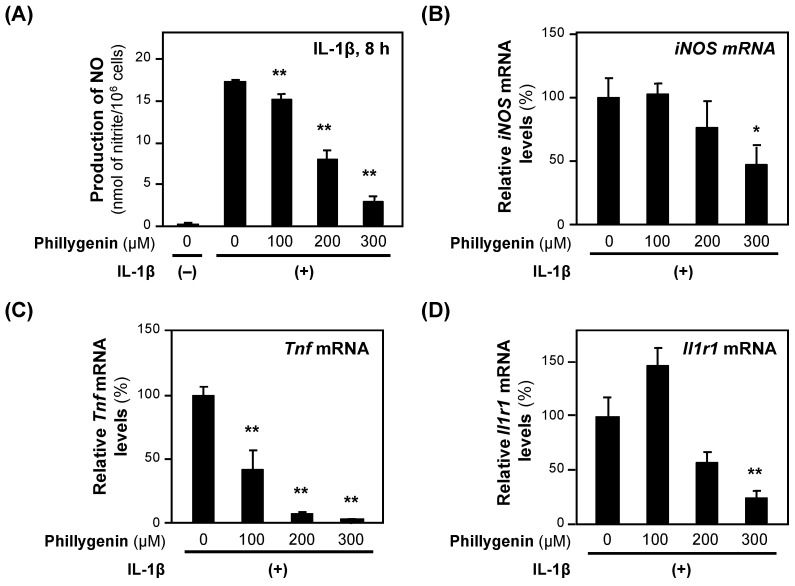
The effects of (+)-phillygenin on the expression of proinflammatory genes. (**A**) A decrease in NO production by (+)-phillygenin in hepatocytes. (+)-Phillygenin and IL-1β were added to the medium of primary-cultured rat hepatocytes and incubated for 8 h until the NO concentration was measured. Cytotoxicity was not observed at the concentrations applied. (**B**–**D**) mRNA levels in hepatocytes. After incubation with (+)-phillygenin and IL-1β, the total RNA was extracted. The level of each mRNA was measured by RT–qPCR and normalized to the *Ef1a* mRNA level. The relative mRNA levels (%) of *iNOS* mRNA (**B**), *Tnf* mRNA (**C**), and *Il1r1* mRNA are presented as the means ± SDs (*n* = 3) when the mRNA level measured was set at 100% in the presence of IL-1β alone. * *p* < 0.05 and ** *p* < 0.01 versus IL-1β alone.

When (+)-phillygenin was added to the medium of the rat hepatocytes together with IL-1β, the NO concentration decreased in a concentration-dependent manner without showing cytotoxicity (Figure 7A). The calculated IC_50_ value of (+)-phillygenin for the suppression of NO production was 193.0 ± 47.6 μM. Like other anti-inflammatory compounds, (+)-phillygenin decreased the levels of *iNOS* mRNA and mRNAs transcribed from proinflammatory genes, such as *Tnf* and *Il1r1* mRNAs (Figure 7B–D). These results indicate that (+)-phillygenin is an anti-inflammatory compound.

## 3. Discussion

Empirical pharmacological function is an important concept in Kampo medicine. In this study, we investigated 10 typical crude drugs for clearing heat and demonstrated that the empirical function of clearing heat involves many anti-inflammatory compounds. Inflammation is characterized by four cardinal signs, i.e., calor (heat), dolor (pain), rubor (redness), and tumor (swelling) [29], and the anti-inflammatory compounds attenuate inflammatory responses, resulting in the cooling of the body.

During the purification of the compounds from Fraction A, we selected the most active subfraction, i.e., one that prominently suppressed NO production. Consequently, we identified constituents that are responsible for anti-inflammatory effects, such as baicalein in *Scutellaria* roots [30,31] and (+)-phillygenin in *Forsythia* fruits [32]. The isolation of loniflavone from *Lonicera japonica* (stems and leaves collected in China) is second, followed by the first isolation from *Lonicera japonica* (leaves collected in India) [33]. We first reported the anti-inflammatory effects of loniflavone in this study. Anti-inflammatory compounds may be isolated from Fraction A of *Rehmannia* roots, when a large amount of plant material is used for the purification. At least several anti-inflammatory compounds are expected to be present in Fraction A. The contribution of each compound to the suppression of NO production depends on its IC_50_ value and content in the extract.

Bioactive compounds are not always major constituents of a crude drug. Baicalin (a baicalein glucuronide), which is present in Fraction B of the *Scutellaria* root extract, reduced NO production, similar to the baicalein in Fraction A. Baicalin had an IC_50_ value of 55.0 μM, which was 3.3-fold greater than that of baicalein in IL-1β-treated hepatocytes (A.F., K.I., M.N. unpublished data). In contrast, geniposide (an iridoid glucoside), which is a major constituent primarily present in Fraction B of *Gardenia* fruit extract, did not decrease NO production in IL-1β-treated hepatocytes. In LPS-treated RAW264.7 cells, geniposide reduced NO production with an IC_50_ value of 136 µM [12], which may be caused by differences in the cell types [14]. Because bacterial β-glucosidase hydrolyzes geniposide to genipin in the intestine [34,35], geniposide functions as genipin in vivo. The bioavailability of glycosides in crude drugs may be improved by the bacterial conversion. Although the amount of each crude drug in a Kampo formula is determined by the *Japanese Pharmacopoeia* [3], effective doses of the compounds in the crude drugs may be investigated in future.

The IC_50_ value of Fraction A from *Forsythia* fruit extract could not be determined because hepatocytes partly detached from the dish bottom, suggesting the cytotoxicity of betulinic and oleanolic acids to hepatocytes (Figure 6). Oleanolic acid is abundant in *Forsythia suspensa* fruits [24], and betulinic acid is widely distributed in plants, such as *Betula platyphylla* and *Ziziphus jujuba* Miller var. *spinosa* Hu ex H. F. Chou. In accordance with our findings, betulinic acid is cytotoxic to tumor cell lines [25,26], and these triterpenoids induced apoptosis in hepatocellular carcinoma cell lines [36,37]. Therefore, small amounts of these triterpenoids co-existing in an extract or Fraction A may mask the anti-inflammatory effects of other compounds, including (+)-phillygenin. Because cytotoxic compounds are sometimes present in crude drugs, separation by silica gel chromatography may help identify the compounds of interest.

When the IC_50_ values of the identified anti-inflammatory compounds for the suppression of NO production in hepatocytes were compared, they presented variable values ranging from 2.6 μM (obakunone) to 652 μM (chlorogenic acid) (Table 3). It is difficult to identify common structures among them. Our previous studies revealed other anti-inflammatory compounds; for example, atractylodin (polyacetylene) from *Atractylodes chinensis* rhizomes inhibited NO production with an IC_50_ value of 8.25 μM in hepatocytes [38]. In contrast, β-eudesmol and (+)-hinesol, which are major constituents of *A. chinensis* rhizome Fraction A, exhibited low activity, and their IC_50_ values could not be calculated.

The compounds identified in this study decreased the expression of the *iNOS* genes, at least at a transcriptional step. Because baicalein reduced the NO production, iNOS protein, and *iNOS* mRNA in hepatocytes (Figure 3), it inhibits the *iNOS* gene expression at a transcriptional step through transcription factors. The other compounds identified in this study and 1,2,3,4,6-pentagalloyl-β-D-glucose [8] reduced the NO production by decreasing both iNOS protein and mRNA (Figure 4, Figure 5 and Figure 7), suggesting they inhibit the *iNOS* gene expression at a transcriptional step. Furthermore, anti-inflammatory compounds, including obakunone [7], chlorogenic acid [9], genipin [10], and baicalein inhibited the *iNOS* gene expression at transcriptional and post-transcriptional steps by reducing *iNOS-AS* [18]. Collectively, the compounds purified from the crude drugs for clearing heat downregulate the *iNOS* gene expression, at least at a transcriptional step.

How do the anti-inflammatory compounds in the crude drugs exert anti-inflammatory effects? Anti-inflammatory compounds or drugs, such as baicalein, attenuate the expression of the *Il1r1* gene (Figure 3). IL-1β induces the expression of the *Il1r1* gene [22], and nonsteroidal anti-inflammatory drugs (NSAIDs), such as aspirin and loxoprofen, suppress *Il1r1* gene expression in rat hepatocytes [14]. NSAIDs modulate the nuclear factor κB (NF-κB) signaling pathway to inhibit the *iNOS* gene expression by reducing *iNOS* mRNA and antisense transcript (*iNOS-AS*) levels [14,18]. Nyasol and phillygenin inhibited inflammatory responses via the NF-κB pathway in the LPS-treated RAW264.7 macrophage line [39,40]. Phillygenin inhibited LPS-induced inflammatory responses through the Toll-like receptor 4 (TLR4)–NF-κB signaling pathway in the human hepatic stellate cell line LX-2 [41]. Furthermore, when the nonalkaloid fraction of *Phellodendron* bark, which includes the strong anti-inflammatory compound obakunone, was administered to healthy mice, prominent decreases in *iNOS* and *Il1r1* mRNA levels were observed in their livers [7]. Baicalein showed anti-inflammatory effects by inhibiting the translocation of the phosphorylated p65 subunit of NF-κB to the nucleus [42,43]. Baicalein attenuated rat liver injuries caused by ischemia–reperfusion and partial hepatectomy by suppressing inflammatory responses through NF-κB and Akt signaling [44]. Sakuranetin [(*S*)-(–)-4′,5-dihydroxy-7-methoxyflavanone], which is an anti-inflammatory compound of cherry bark (a crude drug of Kampo medicines), exhibited similar effects. Sakuranetin decreases both *Il1r1* gene expression and the phosphorylation of the activator isoforms of the transcription factor CCAAT/enhancer-binding protein β (C/EBPβ), which synergistically activates the transcription of the *iNOS* gene with NF-κB [22]. Because IL1R1 is a key protein that regulates proinflammatory genes, repressing the expression of the *Il1r1* gene by anti-inflammatory compounds results in decreased proinflammatory responses.

Our method using hepatocytes is one of the in vitro approaches to examine anti-inflammatory effects of the crude drugs for clearing heat, although it is difficult to elucidate their multiple interacting mechanisms or synergies [45]. Animals may be used to investigate various anti-inflammatory mechanisms. The administration of non-alkaloid fractions of *Phellodendron* bark and *Coptis* rhizome downregulated a variety of genes that are involved in inflammation [7], such as activating transcription factor 2 (Atf2) [46], Krüppel-like factor 10 (Klf10) [47], and suppressor of cytokine signaling 3 (Socs 3) [48]. They are key molecules in the signaling pathways, other than the NF-κB pathway, and may modulate inflammatory responses and interacting mechanisms. In addition, isocoumarines inhibited the enzymes in the leukotriene and prostaglandin pathways [49,50]. Therefore, compounds of the crude drugs for clearing heat might affect various biological activities, including anti-oxidant, antimicrobial, neuroprotective, and anticancer activities.

*A. chinensis* rhizomes are classified as crude drugs that circulate fluid retention (‘*Risui*’) [1,5,6], and this empirical function is closely related to the treatment of kidney disease. The administration of *A. chinensis* rhizome Fraction A to high immunoglobulin A (HIGA) mice [51], a model of human immunoglobulin A (IgA) nephropathy, prominently reduced IgA deposition in renal glomeruli [38]. Atractylodin, β-eudesmol, and (+)-hinesol are present in this Fraction A and may improve the glomerular IgA deposition [38]. Therefore, the empirical function of circulating fluid retention cannot be simply explained by anti-inflammatory effects alone.

An empirical function classified as ‘*Hoketsu*’ involves replenishing blood to improve blood deficiency [1,5,6]. *Polygonum multiflorum* roots are classified as a crude drug that replenishes blood. We found that this crude drug promoted the renal production of erythropoietin, which stimulates red blood cell production in healthy mice [52], which may explain its empirical function in replenishing blood. There are many other empirical functions in the concept of Kampo medicine, such as *Hoki* (reinforcing qi to improve qi deficiency). Here, we elucidate aspects of the empirical function *Seinetsu*. Using other systems or model animals, the empirical functions of Kampo medicine may be explained by modern pharmacological functions in the future.

## 4. Materials and Methods

### 4.1. General Experimental Procedures and Reagents

The NMR spectra were recorded using a JNM-ECS400 NMR spectrometer (JEOL Ltd., Akishima, Tokyo, Japan), which was operated at 400 MHz (^1^H) and 100 MHz (^13^C). Deuterated chloroform (CDCl_3_), methanol (CD_3_OD), dimethyl sulfoxide [(CD_3_)_2_SO], and tetramethylsilane (internal standard) were purchased from Eurisotop (Saint-Aubin, France). The EI–MS spectra were obtained with a JMS-700 MStation mass spectrometer (JEOL Ltd.). The optical rotations of the compounds were measured using a DIP-1000 polarimeter (JASCO Corporation, Hachioji, Tokyo, Japan). The standards baicalein, genipin, geniposide, and betulinic acid were purchased from Tokyo Chemical Industry Co., Ltd. (Tokyo, Japan), and the oleanolic acid 4-methoxybenzaldehyde (=*p*-anisaldehyde) was purchased from Fujifilm Wako Pure Chemical Corporation (Osaka, Japan).

### 4.2. Plant Materials

The crude drugs, authenticated by Dr. Yutaka Yamamoto (Tochimoto Tenkaido Co., Ltd., Osaka, Japan) were obtained from Tochimoto Tenkaido Co., Ltd., and the voucher samples were deposited in the Ritsumeikan Herbarium of Pharmacognosy, Ritsumeikan University, under the following code numbers. *Gardenia jaminoides* fruits were collected in Zhejiang Province, China (No. RIN-GJ-46), *Scutellaria baicalensis* roots were collected in the Inner Mongolia Autonomous Region, China (No. RIN-SB-52), *Anemarrhena asphodeloides* rhizomes were collected in Hebei Province, China (No. RIN-AS-53), *Forsythia suspensa* fruits were collected in Shaanxi Province, China (No. RIN-FS-54), the leaves and stems of *Lonicera japonica* were collected in Shandong Province, China (No. RIN-LJ-55), and *Rehmannia glutinosa* roots were collected in Shaanxi Province, China (No. RIN-RG-56).

### 4.3. Extraction and Crude Fractionation

The dried crude drugs were extracted with methanol and the resulting extracts were successively extracted with EtOAc and *n*-butanol to obtain EtOAc-soluble (Fraction A), *n*-butanol-soluble (Fraction B), and water-soluble fractions (Fraction C), as previously described [9] (Scheme 1). The yield of the extracts and the partitioning ratios of the crude fractions are shown in Table 1.

### 4.4. Purification of Anti-Inflammatory Compounds

The compounds were purified from Fraction A using several methods guided by NO assays in hepatocytes. Silica gel column chromatography was performed with silica gel 60 (Nacalai Tesque Inc., Kyoto, Japan) or Wakogel C-300 HG (Fujifilm Wako Pure Chemical Corporation). PLC silica gel 60 F_254_, 0.5 mm glass plates (Merck KGaA, Darmstadt, Germany) were used to perform preparative TLC. The subfraction that suppressed the NO production was further purified to obtain compounds. The details are described in Supplementary Materials Data, S1.

### 4.5. Measuring the Content of Compounds

The content of each compound was estimated using HPLC according to a previously published method [22], HPLC analysis was performed to estimate the content of compounds. An HPLC system with an LC-20AT pump equipped with an SPD-20A UV/VIS detector (Shimadzu Corporation, Kyoto, Japan) and a Cosmosil 5C_18_ MS-II column (4.6 mm internal diameter × 150 mm; Nacalai Tesque Inc.) was used. The details are described in Supplementary Materials Data, S2.

### 4.6. TLC and p-Anisaldehyde–Sulfuric Acid Color Reactions

The constituents were developed on a silica gel 60 F_254_ plate (Fujifilm Wako Pure Chemical Corporation). The plate was sprayed with a solution prepared from 2.6 mL *p*-anisaldehyde in 1.6 mL glacial acetic acid, 3.4 mL 97% sulfuric acid, and 92 mL ethanol and heated to 105 °C for 10 min to visualize spots of phenols, sugars, steroids, and terpenoids.

### 4.7. Animals and Primary-Cultured Rat Hepatocytes

Specific pathogen-free male Wistar rats (5–6 weeks old, Charles River Laboratories Japan, Inc., Yokohama, Japan) were purchased and maintained at 21–23 °C under a 12 h light–dark cycle. A γ-ray-irradiated CRF-1 diet (Charles River Laboratories Japan, Inc.) was provided with water available ad libitum. The acclimatization time was at least one week. All procedures involving animal care and experiments were performed in accordance with the laws and guidelines of the Japanese government and were approved by the Animal Care Committee of Ritsumeikan University, Biwako-Kusatsu Campus under Nos. BKC2020-045 and BKC2023-052.

After acclimatization, the Wistar rats were euthanized by the intraperitoneal administration of an anesthetic combination of medetomidine hydrochloride, midazolam, and butorphanol tartrate. Rat livers were used to prepare hepatocytes as previously described [53,54]. In brief, the liver was perfused with collagenase. The cells were resuspended in Williams’ E (WE) medium supplemented with 10% newborn calf serum, 10 nM dexamethasone, and 10 nM human insulin and seeded at 1.2 ×10^6^ cells per 35 mm diameter dish. After the medium was replaced with serum-free WE medium, the cells were incubated overnight. The next day, the medium was replaced with WE medium containing 1 nM recombinant rat IL-1β [54] and/or a fraction or compound. The hepatocytes were further incubated at 37 °C for 4 h before RNA extraction or for 8 h before measuring the NO levels and performing a Western blot analysis of the cell lysates.

### 4.8. NO Assay and LDH Activity

After 8 h of incubation with 1 nM IL-1β and/or a fraction or compound, nitrite (a stable metabolite of NO) was measured in triplicate using the Griess method [14,55]. The NO concentration in the medium alone was set at 0%, whereas the concentration in the medium including IL-1β alone was set at 100%. Unless a fraction or compound exhibited cytotoxicity, the IC_50_ values of nitrite were calculated for three different concentrations [14]. Cytotoxicity was monitored by estimating the LDH activity in the medium using LDH Cytotoxicity Detection Kits (Takara Bio Inc., Kusatsu, Japan). The Griess reagent [14,55] was added to the medium and incubated at 20–23 °C for 5 min, and the absorbance at 540 nm was measured to determine the decrease in nitrite. The LDH activity of the whole-cell extract was assumed to be 100%, whereas the LDH activity of the medium alone was assumed to have no cytotoxicity (i.e., less than 5% of the LDH activity of the whole-cell extract).

### 4.9. Immunoblot Analysis

Hepatocyte lysates were prepared, resolved on a 10% sodium dodecyl sulfate–polyacrylamide gel, and transferred onto a membrane, as previously described [22]. After the samples were blocked with 5% skim milk, immunostaining was performed with antibodies against iNOS (BD Biosciences, San Jose, CA, USA) and β-tubulin, and with a horseradish peroxidase-conjugated anti-immunoglobulin Fc antibody (Cell Signaling Technology Inc., Danvers, MA, USA). The protein of interest was visualized by ECL Western blotting Detection Reagents (Cytiva, Tokyo, Japan) and detected using an Amersham Imager 600 (Cytiva).

### 4.10. RT–qPCR

Hepatocytes were lysed in Sepasol I Super G solution (Nacalai Tesque, Inc.) to extract total RNA, which was purified using an RNAqueous Kit and a TURBO DNA-free Kit (Applied Biosystems, Austin, TX, USA). The resulting RNA was converted to cDNA, which was amplified by PCR in triplicate with primers [54], SYBR Green I, and the Thermal Cycler Dice Real-Time System (Takara Bio Inc.) [18]. Ct values were calculated by the ΔΔCt method and then normalized to elongation factor 1α (*Ef1a*) mRNA [18,54]. The mRNA levels normalized to the total RNA from the livers of the mice fed a diet alone were set at 100%.

### 4.11. Statistical Analysis

The data are representative of at least three independent experiments that provided similar findings. The values are presented as the means ± SDs. The differences were analyzed using Student’s *t* test followed by the Bonferroni correction. The statistical significance was set at 0.05 or 0.01.

## 5. Conclusions

Based on the concepts of Kampo medicine, 10 typical crude drugs for clearing heat were examined for their anti-inflammatory effects. The EtOAc-soluble fraction from the crude drugs, except *Forsythia* fruits, exhibited anti-inflammatory effects, and anti-inflammatory compounds were identified from these extracts, with the exception of that from *Rehmannia* roots. Through the removal of cytotoxic compounds, the anti-inflammatory compound (+)-phillygenin was purified from Fraction A of the *Forsythia* fruit extract. Our approach may support the idea that anti-inflammatory compounds in crude drugs provide the empirical function of clearing heat to cool the body. Other empirical functions of Kampo medicine may also be explained by modern pharmacological activities in the future.

## Figures and Tables

**Table 1:** Typical crude drugs for clearing heat ^1^.

Crude Drug	Japanese Name	Description	Family
*Phellodendron* bark	*Obaku*	Bark of *Phellodendron amurense* Ruprecht, or *P. chinense* Schneider	Rutaceae
*Coptis* rhizome	*Oren*	Rhizome of *Coptis japonica* Makino, *C. chinensis* Franchet, *C. deltoidea* C.Y. Cheng et Hsiao, or *C. teeta* Wallich	Ranunculaceae
*Paeonia suffruticosa* root bark (moutan bark)	*Botanpi*	Root bark of *Paeonia suffruticosa* Andrews	Paeoniaceae
*Lonicera* flower	*Kinginka*	Flower and bud of *Lonicera japonica* Thunberg	Caprifoliaceae
*Gardenia* fruit	*Sanshishi*	Fruit of *Gardenia jasminoides* Ellis	Rubiaceae
*Scutellaria* root	*Ogon*	Root of *Scutellaria baicalensis* Georgi	Labiatae
*Anemarrhena* rhizome	*Chimo*	Rhizome of *Anemarrhena asphodeloides* Bunge	Liliaceae
*Lonicera* leaf and stem	*Nindo*	Leaf and stem of *Lonicera japonica* Thunberg	Caprifoliaceae
*Rehmannia* root	*Jio*	Root of *Rehmannia glutinosa* Liboschitz var. *purpurea* Makino, or *R. glutinosa* Liboschitz	Scrophulariaceae
*Forsythia* fruit	*Rengyo*	Fruit of *Forsythia suspensa* Vahl	Oleaceae

^1^ Selected from the lists [3,5,6].

**Table 2:** The extraction and fractionation of crude drugs for clearing heat.

**Crude Drug**	**Extraction** **Yield (%) ^1^**	**Fraction A**		**Fraction B**		**Fraction C**		**Reference**
		**Ratio (%) ^2^**	**IC_50_ (µg/mL) ^3^**	**Ratio (%) ^2^**	**IC_50_ (µg/mL) ^3^**	**Ratio (%) ^2^**	**IC_50_ (µg/mL) ^3^**	
*Phellodendron* bark	19.7	--- *		--- *		--- *		[7]
*Coptis* rhizome	18.0	--- *		--- *		--- *		[7]
Moutan bark	25.9	22.2	8.97 ± 0.06	9.93	141 ± 23.5	67.9	NA	[8]
*Lonicera* flower	34.5	11.1	126 ± 30	16.4	993 ± 327	72.5	>2000	[9]
*Gardenia* fruit ^4^	24.3	29.6	231 ± 20.3	41.4	NA	29.0	NA	This study
*Scutellaria* root	34.6	15.9	8.60 ± 2.07	22.5	55.3 ± 27.3	61.7	122 ± 24.3	This study
*Anemarrhena* rhizome	26.9	13.9	42.8 ± 7.56	24.3	NA	61.8	NA	This study
*Lonicera* leaf and stem	15.8	19.6	37.7 ± 3.16	29.5	233 ± 24.3	50.9	NA	This study
*Rehmannia* root	18.1	1.71	18.1 ± 2.83	5.68	128 ± 41.1	92.6	573 ± 247	This study
*Forsythia* fruit	8.93	58.0	NC	28.0	192 ± 13.9	14.0	556 ± 51.0	This study

^1^ (dry weight of methanol extract)/(dry weight of starting plant material) × 100. ^2^ The percentage was calculated as the weight of each fraction divided by the sum of three fractions. ^3^ The half-maximal inhibitory concentration for NO production in hepatocytes in the presence of IL-1β (mean ± SD). ^4^ Values were determined in this study. * Fractionated by another method. NA, not applicable because of its low effect on NO production. NC, not applicable owing to high cytotoxicity (see text).

**Table 3:** The IC_50_ values of anti-inflammatory compounds in crude drugs for clearing heat.

Crude Drug	Typical Compound	Classification	IC_50_ (μM) ^1^	Reference
*Phellodendron* bark	Obakunone	Limonoid	2.6 ± 1.1	[7]
*Coptis* rhizome	Berberine	Alkaloid	12.0 ± 3.4	[7]
Moutan bark	1,2,3,4,6-Pentagalloyl-β-D-glucose	Tannin	2.67 ± 0.36	[8]
*Lonicera* flower	Chlorogenic acid	Phenylpropanoid	652 ± 114	[9]
*Gardenia* fruit	Genipin	Iridoid	4.40 ± 0.85	This study
*Scutellaria* root	Baicalein	Flavonoid	16.8 ± 7.93	This study
*Anemarrhena* rhizome	(−)-Nyasol	Phenylpropanoid	18.3 ± 2.99	This study
*Lonicera* leaf and stem	Loniflavone	Flavonoid	1.64 ± 0.17	This study
*Forsythia* fruit	(+)-Phillygenin	Lignan	193 ± 47.6	This study

^1^ The half-maximal inhibitory concentration for NO production in IL-1β-stimulated hepatocytes. At least three experiments were performed to determine these values (means ± SDs).

## Data Availability

The data presented in this study are available on request from the corresponding author.

## Supplementary Materials

The following supporting information can be downloaded at: https://www.mdpi.com/article/10.3390/molecules30020416/s1, Purification of anti-inflammatory compounds from crude drugs. Measuring the content of compounds.
