# Supplementary material for: Crude Drugs for Clearing Heat Contain Compounds Exhibiting Anti-Inflammatory Effects in Interleukin-1β-Treated Rat Hepatocytes
Source: Molecules. 2025 Jan 19;30(2):416. doi: 10.3390/molecules30020416 (PMC11768083; doi:10.3390/molecules30020416)
Supplement: Supplementary file 1 [file molecules-30-00416-s001.zip › molecules-3395668-supplementary.pdf]

Supplementary Data

# Crude Drugs for Clearing Heat Contain Compounds Exhibiting Anti-Inflammatory Effects in Interleukin-1β-Treated Rat Hepatocytes

**Airi Fujii [1], Saki Onishi [1], Nodoka Watanabe [1], Mizuki Kajimura [1], Kentaro Ito [1], Keita Minamisaka [1], Yuto Nishidono [2], Saki Shirako [1], Yukinobu Ikeya [2,3] and Mikio Nishizawa [1,4,*]**

[1] Department of Medical Biosciences, Faculty of Life Sciences, Ritsumeikan University, Kusatsu 525-8577, Shiga, Japan; fujii-ai@fc.ritsumei.ac.jp (A.F.); t-onishisaki@medu.pref-osaka.ed.jp (S.O.); n.watanabe0108@gmail.com (N.W.); mizuki.1100.moon.13.1098@gmail.com (M.K.); kn.ito1005@gmail.com (K.I.); si0078kk@ed.ritsumei.ac.jp (K.M.); sshirako@fc.ritsumei.ac.jp (S.S.)
[2] Research Organization of Science and Technology, Ritsumeikan University, Kusatsu 525-8577, Shiga, Japan; nisidono@fc.ritsumei.ac.jp (Y.N.); y-ikeya@daiichi-cps.ac.jp (Y.I.)
[3] Faculty of Pharmacy, Daiichi University of Pharmacy, Fukuoka 815-8511, Fukuoka, Japan
[4] Department of Biology, Faculty of Mathematics and Natural Sciences, Universitas Brawijaya,
Malang 65113, East Java, Indonesia
* Correspondence: nishizaw@sk.ritsumei.ac.jp; Tel.: +81-77-561-2876

## Contents

**S1. Purification of anti-inflammatory compounds from crude drugs**

*S1.1. Purification of baicalin from Scutellaria roots*

*S1.2. Purification of nyasol from Anemarrhena rhizomes*

*S1.3. Purification of loniflavone from Lonicera leaves and stems*

*S1.4. Purification of phillygenin from Forsythia fruits*

**S2. Measuring the content of compounds**

*S2.1. Measurement of the content of baicalin*

*S2.2. Measurement of the content of nyasol*

**S3. References**

**S1. Purification of anti-inflammatory compounds from crude drugs**

*S1.1. Purification of baicalein from Scutellaria roots*

Fraction A (19.0 g) was obtained from the *Scutellaria* root extract (138 g) using a previously published method [1]. Fraction A (193 mg) was subjected to Sephadex LH-20 column chromatography [1.0 cm internal diameter (i.d.) × 15 cm], and the compounds were separated using methanol:water (1:1) to obtain subfractions A1–A6. Subfractions A2 to A4 were developed using preparative thin-layer chromatography (TLC) with *n*-hexane:acetone (2:1) to obtain compound of a yellow powder (5.14 mg), which was designated as Compound **1** and identified as baicalein, as described in the text.

*S1.2. Purification of nyasol from Anemarrhena rhizomes*

Fraction A (14.7 g) was obtained from *Anemarrhena rhizome* extract (275 g) [1]. Fraction A (10.1 g) was separated using silica gel column chromatography. The compounds were eluted stepwise using *n*-hexane:ethyl acetate (EtOAc) (100:0 to 2:1) to give subfractions A1 to A14. Subfraction A9, which inhibited NO production in IL-1β-treated hepatocytes, was subjected to preparative TLC using *n*-hexane:EtOAc (1:1) to obtain Compound **2** (18.5 mg), which was identified as (−)-nyasol, as described in the text.

*S1.3. Purification of loniflavone from Lonicera leaves and stems*

Fraction A (24.8 g) was obtained from the extract (127 g) of *Lonicera leaves and stems* [1]. Fraction A (24.8 g) was separated by silica gel column chromatography, and the compounds were eluted stepwise using chloroform:acetone (100:0 ---> 0:100) to yield subfractions A1–A6. Subfraction A3, which inhibited NO production in IL-1β-treated hepatocytes, was further purified by silica gel column chromatography and preparative TLC using EtOAc:chloroform:acetone (3:3:4) to obtain Compound **3** (5.73 mg), which was identified as loniflavone, as described in the text.

*S1.4. Purification of phillygenin from Forsythia fruits*

Fraction A (26.6 g) was obtained from the *Forsythia* fruit extract (45.8 g) [1]. Fraction A (26.6 g) was separated with silica gel column chromatography (6.5 cm i.d. × 30 cm), and the compounds were eluted stepwise with *n*-hexane:EtOAc (100:0 ---> 0:100) to produce subfractions LA1–LA13. Subfraction LA8 was purified by silica gel column chromatography using chloroform:acetone (95:5 ---> 50:50) to obtain 11 subfractions, LA8-1 to LA8-11. One of the subfractions that inhibited NO production in IL-1β-treated hepatocytes, was further purified to obtain Compound **4** (174 mg), which was identified as (+)-phillygenin, as described in the text.

**S2. Measuring the content of compounds by HPLC**

*S2.1. Measurement of the content of baicalein*

HPLC analysis was performed to estimate the content of Compound **1**, i.e., baicalein, according to a previously published method [2]. An HPLC system with an LC-20AT pump equipped with an SPD-20A UV/VIS detector (Shimadzu Corporation, Kyoto, Japan) and a Cosmosil 5$C_{18}$ AR-II column (4.6 mm i.d. × 150 mm; Nacalai Tesque Inc.) was used. The compounds were eluted at a flow rate of 1.0 mL/min with a 20 mM phosphoric acid:methanol mixture (45:55) and detected at a wavelength of 280 nm. Single peaks with a retention time of 12.3 min corresponded to baicalein. The retention time was used to estimate the baicalein content in Fraction A. Isolated baicalein was used as a standard by accurately weighing and dissolving it in methanol to prepare a stock solution of 1.24 mg/mL. Each stock solution was diluted to prepare standard solutions at the following concentrations: 0.31, 0.62, and 1.24 mg/ml. Each standard solution (10.0 μL) was analyzed in triplicate. The calibration curve of each standard compound was calculated by plotting the peak areas ($y$) against a series of injection amounts ($x$), and the regression equation was calculated in the form $y$ = A$x$ + B: $y$ = 58,509,702 $x$ – 547,109 ($R^2$ = 0.9984). Fraction A was accurately weighed and dissolved in methanol to prepare a sample solution of 2.53 mg/mL, and the sample solution (10.0 μL) was analyzed in triplicate. The peak areas of the compounds in the sample solution were fitted to the calibration curves, and the amounts of baicalein in 10.0 μL of the sample solution were calculated. The amount of the compounds in 10.0 μL of the sample solution (25.3 μg of fraction A) was calculated to be 5.7 μg. Therefore, the content of baicalein in Fraction A was 22.5%.

*S2.2. Measurement of the content of nyasol*

HPLC analysis was performed to estimate the content of Compound **2**, i.e., (−)-nyasol [2] using an HPLC system with a Cosmosil 5$C_{18}$ AR-II column (Nacalai Tesque Inc.). (−)-Nyasol was eluted at a flow rate of 1.5 mL/min with a 0.1% formic acid:acetonitrile mixture (60:40) and detected at a wavelength of 302 nm. Single peaks with a retention time of 8.92 min corresponded to (−)-nyasol. The retention time was used to estimate its content in Fraction A. Isolated (−)-nyasol was used as a standard by accurate weighing and dissolving it in acetonitrile to prepare a stock solution of 1.00 mg/mL. Each stock solution was diluted to prepare standard solutions at the following concentrations: 0.5, 1.0, and 2.0 mM (−)-nyasol. Each standard solution (20.0 μL) was analyzed in triplicate. The calibration curve of each standard compound was calculated by plotting the peak areas ($y$) against a series of injection amounts ($x$), and the regression equation was calculated in the form $y$ = A$x$ + B: $y$ = 477,694.0 $x$ – 48,542.86 ($R^2$ = 0.9993). Fraction A was accurately weighed and dissolved in methanol to prepare a sample solution of 5.00 mg/mL, and the sample solution (20.0 μL) was analyzed in triplicate. The peak areas of the compounds in the sample solution were fitted to the calibration curves, and the amounts of (−)-nyasol in 20.0 μL of the sample solution were calculated. Therefore, the content of (−)-nyasol in fraction A was 5.56%.

**S3. References**


1. Ohno, N.; Yoshigai, E.; Okuyama, T.; Yamamoto, Y.; Okumura, T.; Sato, K.; Ikeya, Y.; Nishizawa, M. Chlorogenic acid from the Japanese herbal medicine Kinginka (Flos Lonicerae japonicae) suppresses the expression of inducible nitric oxide synthase in rat hepatocytes. *HOAJ Biol*. **2012**, 1, 2. https://doi.org/10.7243/2050-0874-1-2
2. Yamauchi, Y.; Okuyama, T.; Ishii, T.; Okumura, T.; Ikeya, Y.; Nishizawa, M. Sakuranetin Downregulates Inducible Nitric Oxide Synthase Expression by Affecting Interleukin-1 Receptor and CCAAT/Enhancer-Binding Protein β. *J Nat Med* **2019**, 73. https://doi.org/10.1007/s11418-018-1267-x
